# 618 Firefighter Resiliency Project: Survey Findings and Implications for a Program Model

**DOI:** 10.1093/jbcr/irac012.246

**Published:** 2022-03-23

**Authors:** Dana R Dillard, Erin A King, Kellie O'Dare, Rob Rotunda

**Affiliations:** University of West Florida, Pensacola, Florida; Department of Social Work, University of West Florida, Pensacola, Florida; FL A & M Univ, TALLAHASSEE, Florida; Univ of West Florida, Pensacola, Florida

## Abstract

**Introduction:**

Firefighters are trained to respond to an array of acute emergencies that culminate in repeated exposure to trauma subsequently impacting their mental health. Yet, they often fail to seek services that could mitigate those adverse effects due to stigma, as well as a dearth of systematized resources and first responder proficient trained mental health professionals. This study explored work-related traumatic event exposure, PTSD symptomatology, levels of depression, anxiety, and suicide risk, and barriers to care for firefighters in a designated catchment area of a southeastern state. Results were used to inform local departments interested in culture change and intervention, as well as providing the basis for successfully obtaining federal grant monies.

**Methods:**

Using Qualtrics, a web-based survey platform, researchers administered a cross-sectional survey to firefighters between Fall 2020 and Spring 2021. The survey included the PCL-5, PHQ-9, GAD-7, SBQ-R, and BACE. Descriptive statistics, correlations, and independent t-tests were run to determine the level of trauma exposure and clinically significant mental health symptomatology, associations between different types of trauma and mental health symptoms, and barriers to accessing care. The sample (N=511) primarily identified as Caucasian (n=421) and male (n=477). Mean age and time in fire service were 39.1 and 14.5 years, respectively.

**Results:**

In this sample, 18.7% met the criteria for a provisional diagnosis of PTSD based on the PCL-5; 24.4% met the criteria for moderate to severe depression based on the PHQ-9; 14.5% met the criteria for moderate to severe anxiety based on the GAD-7; and 13.7% reported a significant risk of suicidal behavior as measured by the SBQR. Firefighters also indicated the following as the most common barriers to accessing care: 1) being unsure of where to get care (47.3%); 2) thinking the problem would get better itself (41.8%), 3) feeling embarrassed or ashamed (39%), 4) concern that they might be seen as weak for having a mental health problem (36.2%), and 5) thinking they did not have a problem (34.3%).

**Conclusions:**

Due to the high levels of work-related trauma exposure, firefighters in this study were at an increased risk of developing mental health symptomatology including PTSD, depression, anxiety, and risk of suicidal behavior when compared to the general population. Additionally, perceived and actual barriers to care provided implications for the grant program application.

